# Cellular graphene aerogel combines ultralow weight and high mechanical strength: A highly efficient reactor for catalytic hydrogenation

**DOI:** 10.1038/srep25830

**Published:** 2016-05-12

**Authors:** Bingxing Zhang, Jianling Zhang, Xinxin Sang, Chengcheng Liu, Tian Luo, Li Peng, Buxing Han, Xiuniang Tan, Xue Ma, Dong Wang, Ning Zhao

**Affiliations:** 1 Beijing National Laboratory for Molecular Sciences, CAS Key Laboratory of Colloid and Interface and Thermodynamics, Institute of Chemistry, Chinese Academy of Sciences, China.

## Abstract

The construction of three-dimensional graphene aerogels (GAs) is of great importance owing to their outstanding properties for various applications. Up to now, the combination of ultralow weight and super mechanical strength for GA remains a great challenge. Here we demonstrate the fabrication of cellular GAs by a facile, easily controlled and versatile route, i.e. the chemical reduction of graphene oxide assemblies at oil-water interface under a mild condition (70 °C). The GA is ultralight (with density <3 mg cm^−3^) yet mechanically resilient because the walls of the cell closely pack in a highly ordered manner to maximize mechanical strength. The GA has been utilized as an appealing reactor for catalytic hydrogenation, which exhibited great advantages such as large oil absorption capability, exceptional catalytic activity, ease of product separation and high stability.

Graphene is known as a single-layer two-dimensional (2D) structure of carbon atoms, which has been widely applied in energy-storage devices, sensors and catalysis^1^,^2^,^3^. Recently, lots of efforts have been devoted to the construction of three-dimensional (3D) aerogels of graphene because of their outstanding properties, such as super electrochemical performance for energy storage^4^,^5^, high surface area for adsorption^6^,^7^ and important role as carrier for catalysis^8^,^9^. Different methods including chemical vapor deposition^10^, leavening^11^, microwave irradiation^12^, solvothermal reaction^5^,^6^ and chemical reduction^13^,^14^,^15^,^16^,^17^ have been successfully applied to the fabrication of graphene aerogels (GAs). However, most GAs present random structures and suffer from poor mechanical properties that cannot meet the needs of practical applications. The involvement of a secondary phase in GA (e.g. metal ions^8^, carbon nanotubes^18^ or polymer^19^) has been proved to be efficient in maintaining the 3D architecture of graphene and enhancing its mechanical strength. Unfortunately, the support in GA unavoidably influences the intrinsic properties of graphene, which is not applicable for its utilization in different fields. It is desirable to achieve the additive-free graphene bulk materials with properties arising from the nature of individual graphene sheets and the monolithic 3D structures.

The cellular structures of polymers, elastomers and metals have been considered to make the lightweight materials mechanically resilient, because the cell walls pack closely in a highly ordered manner to maximize mechanical strength^20^. Most recently, the fabrication of cellular GAs has been sporadically reported via freeze casting^21^, multi-step soft/hard templating^22^ and modified hydrothermal process^23^. Despite the improved mechanical strength of the GAs, the combination of ultralow weight and super mechanical strength still remains a great challenge, which may be due to the difficulty in achieving an ordered cellular structure especially as the density of GA is minimized (e.g. <3 mg cm^−3^). It is urgent and necessary to develop feasible routes for the fabrication of highly ordered cellular GA, through which the marriage between ultralow weight and excellent mechanical strength could be realized.

Here we proposed a novel route for the fabrication of graphene-based cellular aerogels, i.e. by the chemical reduction of graphene oxide (GO) assemblies at oil-water interface under a mild condition (70 °C). This method can produce the cellular structure of GA by a one-pot chemical reduction, which is simpler than the reported multi-step reduction and freeze casting^21^ or the multi-step soft/hard templating method^22^. Moreover, the route proceeding at mild conditions is easily operated and favorable for the preservation of ordered macropores, which is superior to hydrothermal conditions^23^. The cellular GA presents a highly ordered macroporous structure, combining ultralow weight (with densities <3 mg cm^−3^) and super mechanical strength. Owing to the unique features of the as-synthesized GA, it can be used as an appealing reactor for the catalytic hydrogenation with great advantages such as large oil absorption capability, exceptional catalytic activity, ease of product separation and high stability.

## Results

### GA Fabrication

**Figure 1:**
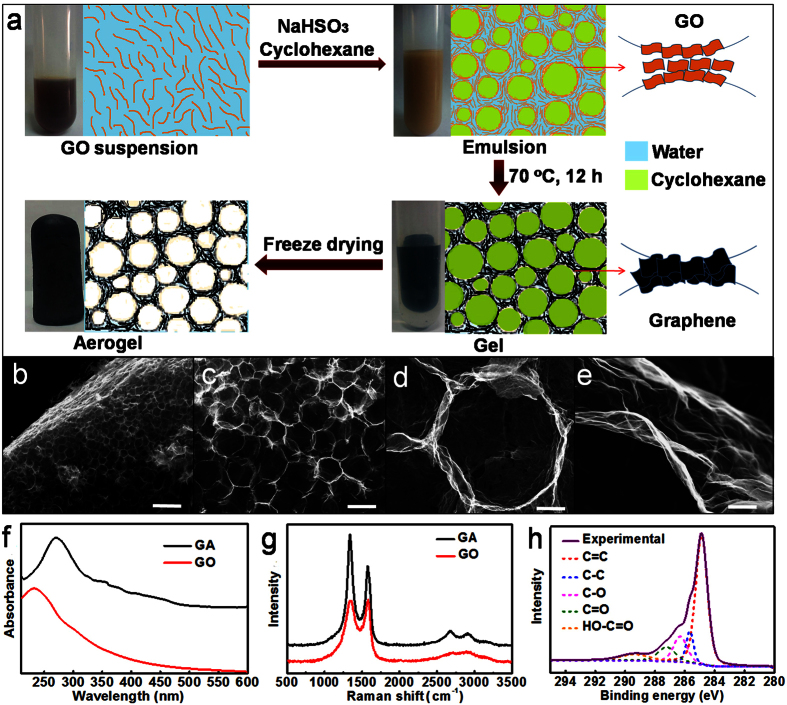
GA fabrication and characterizations. (**a**) Scheme illustrating the synthesis process of GA from the assembly of GO at oil-water interface and the subsequent chemical reduction. (**b**) Whole view of the GA derived from the emulsion with GO concentration of 2.00 mg mL^−1^, revealing that the bulk of aerogel is entirely composed of cellular-like pores. (**c**) The image shows the closely linked pores with polyhedral morphology. (**d**) A hexagonal pore shares the boundary with other six adjacent pores, ensuring the firm bridge of the connection. (**e**) The ultrathin and wrinkled wall. (**f** ) UV-vis spectra of the aqueous suspensions of GO and GA. (**g**) Raman spectra of GO and GA. (**h**) C 1s XPS spectra of GA. Scale bars 150 μm (**b**), 50 μm **(c)**, 8 μm (**d**), and 500 nm (**e**).

The GA fabrication route includes the formation of GO-stabilized emulsion, the mild chemical reduction of GO at oil-water interface and the solvent removing, as illustrated in Fig. 1a. Firstly, the GO flakes with lateral side of several micrometers and thickness of ~1 nm were prepared (see TEM and AFM images in Supplementary Fig. 1). The as-synthesized GO was used to emulsify the cyclohexane/water mixture due to its feasible amphiphilicity^24^ in the presence of NaHSO_3_ under ultrasonication. Then the GO-stabilized emulsion was heated at 70 °C for 12 h to allow the reduction of GO. During this process, the oxygen functional groups in GO sheets were gradually removed, resulting in the reduced hydrophilicity and thus the separation of partial water. The graphene sheets closely stack around the oil droplets to form a honeycomb network, mainly through the hydrophobic and π-π interactions of the conjugated graphene. Finally, the oil and slight water in the fully reduced graphene gel were removed by freeze drying to obtain GA. In comparison with the reported routes for fabricating GA, the strategy proposed here has many unique advantages. First, the GO-stabilized emulsion can be formed at very low GO concentration (e.g. 0.25 mg mL^−1^), which ensures the formation of GA with high porosity and ultralow density. NaHSO_3_ works not only as a reducing agent but also as a co-emulsifier for the GO-stabilized emulsion due to the salt effect^25^ (see the photographs of the emulsions formed with and without NaHSO_3_ in Supplementary Fig. 2). Second, the reduction of GO in emulsion can proceed at a mild temperature (70 °C), which is favorable for the preservation of highly ordered macropores in GA. By contrast, the hydrothermal or solvothermal reduction routes for synthesizing GA monolith usually need high temperature up to 180 °C^5^,^6^,^23^. Third, the two liquids (water and cyclohexane) in graphene gel can be removed simultaneously by freeze drying since they have the similar melting point and the GA was thus directly obtained, simpler than the reported method that oil and water were dividually separated^23^.

Figure 1b–e show the SEM images of the GA derived from the cyclohexane-in-water emulsion with GO concentration of 2.00 mg mL^−1^. The GA has a highly ordered honeycomb-like structure with interconnected macropores, which present a uniform shape of polyhedron and distribute in size of tens of micrometers (Fig. 1b–d). The wall of the macropores is ultrathin and wrinkled (Fig. 1e), different from the smooth sheets of the pristine GO (Supplementary Fig. 1). The as-synthesized GA monolith has a density of 2.8 mg cm^−3^. For comparison, GA was synthesized by reducing the GO dispersed in aqueous solution (in absence of oil and emulsion), while the other experimental conditions being the same with those above. It shows an irregular flocculent structure and no macropores were observed (Supplementary Fig. 3). The density (27.3 mg cm^−3^) is nearly ten times higher than that of the GA obtained from emulsion. It confirms that the emulsion is efficient in conducting the assemblies of GO flakes at oil-water interface and the subsequent formation of cellular GA monolith. Moreover, the as-synthesized GA is better than that obtained via a hydrothermal process (180 °C) from an aqueous emulsion of GO containing hexane droplets^23^, which has disordered macropores and higher density (e.g. 6.73 mg cm^−3^, synthesized under the same GO concentration and 1:2 hexane-to-water volume ratio)^23^. The combination of ultralow weight and ordered macroporous structure of the GA synthesized in this work can be attributed to the unique advantages of the proposed route. The GO-stabilized emulsion with more dispersed phase (1:1 cyclohexane-to-water volume ratio) is favorable for producing a more porous GA structure, while the gentle reduction process at a relatively low temperature and the simultaneous removing of the two liquids by freeze drying guarantee the well preservation of the ordered macroporous structure.

The conjugated structure of the as-synthesized GA was analyzed by different techniques. As seen from UV-vis spectra (Fig. 1f), GO has the maximum absorption peak at 232 nm, corresponding to the π-π^*^ transitions from aromatic C-C bonds^26^. This peak for GA is red-shifted to 271 nm, indicative of the restoration and enhancement of π-electron conjugation^27^. Raman spectra of GO and GA give support for the structure variation after reduction. The characteristic Raman active modes of graphene and other carbon allotropes, represented by G band and D band, in GO sheets are located at 1585 cm^−1^ and 1345 cm^−1^, respectively (Fig. 1g). These two bands are shifted to 1580 cm^−1^ for G band and 1341 cm^−1^ for D band in GA (Fig. 1g). The value of the G band in GA is more close to that of pristine graphite, which also confirms the partial restoration of conjugated structure in GA. Meanwhile, the ratio of the D band and G band intensities increases from 0.98 to 1.36 after the chemical treatment, strongly suggesting that GO was chemically converted to the reduced GO^11^,^28^,^29^,^30^,^31^. Moreover, the GA presents a more pronounced 2D peak at 2680 cm^−1^, compared with that of GO. It further proves that the chemical reduction promotes the recrystallization of graphene^32^. The C 1s XPS spectrum of GA (Fig. 1h) has the following components of carbon bonds: C=C (284.8 eV), C-C (285.6 eV), C-O (hydroxyl and epoxy, 286.3 eV), C=O (287.2 eV) and O-C=O (289.3 eV). Compared with the XPS spectra of GO (Supplementary Fig. 4), the remarkable reduction of oxygen species in GA reveals the significant deoxidization of GO after the chemical reduction. So the sp^2^ domains on the plane of reduced GO increase during the reduction process and the graphene sheets are more likely to aggregate with each other due to the removal of oxygenic functionalities.

### Modulation on GA architectures

**Figure 2:**
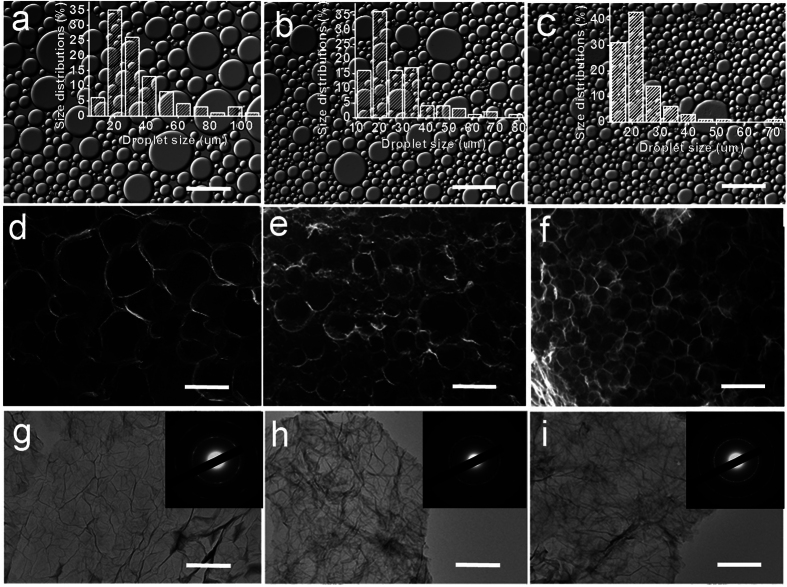
Effect of GO concentration on the microstructures of emulsions and GAs. (**a–c**) CLSM images of the emulsions with GO concentrations of 0.75 mg mL^−1^, 1.25 mg mL^−1^ and 2.00 mg mL^−1^, respectively. Droplet size distributions of the emulsions are shown in the insets. (**d–i**) SEM and TEM images of GA-1 (**d,g**), GA-2 (**e,h**) and GA-3 (**f,i**), derived from the emulsions with GO concentrations of 0.75 mg mL^−1^, 1.25 mg mL^−1^ and 2.00 mg mL^−1^, respectively. The macropore sizes of the three GAs are consistent with the droplet sizes of the respective emulsions. The insets in g-i are the electron diffraction of the cell wall, from which it can be deduced that the thickness of the wall increases with the increasing GO concentration. Scale bars, 100 μm in (**a–f)** and 1 μm in (**g–i)**.

Owing to the tunability of emulsion (e.g. by varying the emulsifier concentration), it is highly attractive to assemble GA architectures with tunable properties in the GO-stabilized emulsions. The effect of GO concentration on the emulsion properties was first investigated. The confocal laser scanning microscopy (CLSM) images of the emulsions stabilized by GO show that the oil droplets are in spherical or polyhedral shape (Fig. 2a–c). With the increasing GO concentration, obviously, the droplet size decreases with a narrower size distribution. It is because more emulsifier (GO sheets) can stabilize the larger interfacial area in emulsion system^25^. The GAs obtained from the emulsions with GO concentrations of 0.75 mg mL^−1^, 1.25 mg mL^−1^ and 2.00 mg mL^−1^ were denoted as GA-1, GA-2 and GA-3, respectively. As shown in Fig. 2d–f, all the three GAs present a 3D honeycomb network. The macropore sizes of the GAs are consistent with the droplet sizes of the respective emulsions (Fig. 2a–c). It confirms that the emulsion can well conduct the assembly of GO and the subsequent reduction of GO to graphene at oil-water interface. The TEM images reveal the folded and crumpled regions along the surfaces of the assembled graphene sheets (Fig. 2g–i), which may contribute to enhancing the mechanical properties of the cell walls^12^. The electron diffraction patterns of the three aerogels indicate that the thickness of the cell wall increases with the increasing GO concentration (insets in Fig. 2g–i)^21^. It is easily understood that more GO sheets assemble at the oil-water interface in emulsion with higher GO concentration. The density of the GA can be lowered to 1.1 mg cm^−3^ by decreasing the GO concentration to 0.75 mg mL^−1^. It is worth noting that as the concentration of GO precursor is as low as 0.25 mg mL^−1^, the aerogel monolith of graphene can also be obtained from the GO-stabilized emulsion. However, the macropores of the GA are disordered (Supplementary Fig. 5), possibly due to the fact that such a small amount of GO sheets is insufficient to support the ordered porous structure during the reduction process.

The XRD patterns show that the wide peak of the GA slightly moves to the higher degree region (from 22.6^o^ to 23.8^o^) in the turn of GA-1, GA-2 and GA-3 (Supplementary Fig. 6), corresponding to a decreased d-spacing of graphene sheets (from 3.90 Å to 3.74 Å). These values are much lower than that of the GO (7.9 Å, Supplementary Fig. 6). It suggests the existence of π-π stacking between graphene sheets in the GA and also the presence of residual oxygenated functional groups on the reduced GO sheets^5^. The XPS results (Supplementary Fig. 7) confirm an effective reduction from GO to graphene. The C/O atomic ratios for the GO, GA-1, GA-2 and GA-3 were calculated from XPS to be 2.78, 5.16, 6.16 and 6.39, respectively (Table 1). Obviously, the deoxidization degree of the GA increases with the increasing GO concentration. It can be partly attributed to the existence of more NaHSO_3_ in the emulsion with higher GO concentration during the reduction process, because it was observed that less amount of water was separated from graphene gel. The porosity degrees of the three GAs are higher than 95% and increase with the decreasing GO concentration, as determined by mercury porosimetry method. Particularly, the porosity of GA-1 can reach 98.6% with an ultrahigh pore volume of 130.0 cm^3^ g^−1^. The macropore size distributions of the three GAs (Supplementary Fig. 8) reveal the decreased macropore size with the increasing GO concentration, consistent with the SEM results shown in Fig. 2d–f. All these data are listed in Table 1. Especially, the Yong’s modulus of GA-3 is among the highest values of the GAs reported with a similar or higher density (Supplementary Table 1).

### Mechanical behavior of GA

**Figure 3:**
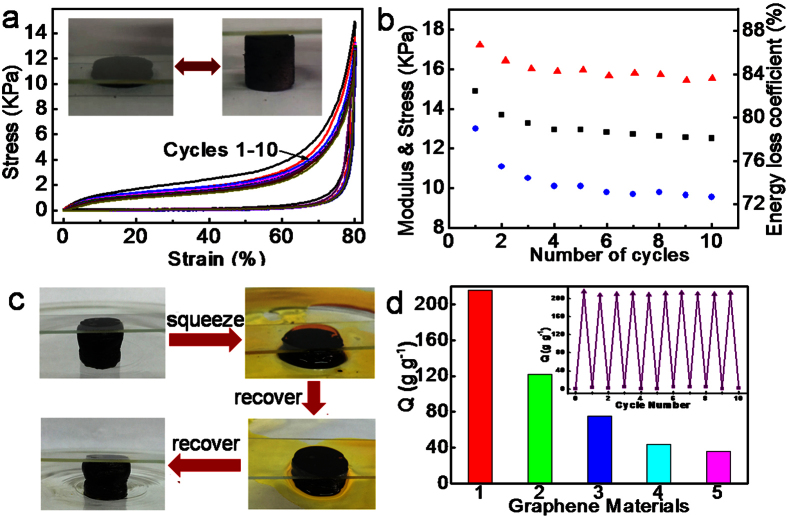
Compression and oil absorption tests of GA-3. (**a**) Stress-strain curves of compressions of the 10 cycles on GA-3, exhibiting elastic deformation. Inset, photographs of the GA under a compressing and releasing cycle. (**b**) The corresponding Young’s modulus (blue circles), retention of maximum stress (black squares) and energy loss coefficient (red triangles) for different cycles obtained from (**a**). (**c**) Recycling absorption approach for GAs absorbers, showing excellent repeated absorption of n-hexane (dyed with Sudan orange G). (**d**) Comparison of n-hexane absorption capacities (Q) for different graphene based materials: (1) GA-3 synthesized in this work, (2–5) graphene sponges reported in ref. 7, 40, 41 and 11, respectively. The inset shows absorption recyclability of GA-3 for n-hexane.

The compression test was performed on the as-synthesized GA. The stress-strain curves of compressions on GA obviously show the typical characteristics of honeycomb-like foam (Fig. 3a)^20^. The structure during the compression process exhibits first a linear elastic region (<5%) corresponding to cell wall bending and cell face stretching, followed by a wide plateau in the range 5–70%, which can be ascribed to cell collapse by elastic buckling^20^. The final region of rapidly increasing stress (70–80%) is typically associated with the densification of cells because of the close touch between opposing cell walls of the almost completely collapsed cell. The maximum stress at 80% in the first cycle can reach 14.8 KPa, which is about two times higher than that of the cork-like GA at the similar density (7.5 KPa for the GA with a density of 2.83 mg cm^−3^)^21^ and much higher than other carbon-based foams with a similar or higher density^7^,^33^. It means that the GA can bear over 70,000 times of its own weight at this strain. During the unloading process, the stress decreases fast in the beginning because most adsorbed energy from the compression process is dissipated due to the buckling of microstructures, friction, fractures and branches adhesion in the resilient cellular structure^34^. As a result, an obvious hysteresis loop was observed after a loading-unloading cycle, similar to other resilient cellular foam^20^,^21^. Remarkably, the tenth cycle curve is similar to the first cycle, implying excellent elastic recovery of the GA. It is interesting that although no abundant energy contributes to the recovery of original shape, the aerogel still can repeatedly recover fast from the over 80% deformation. As reflected from Fig. 3a and Supplementary Movie 1, no volume shrinkage was observed for the aerogel monolith after the loading was removed.

The excellent elasticity of the as-synthesized GA monolith can also be confirmed from the stability of the cyclic resilient property shown in Fig. 3b. The corresponding Young’s modulus, maximum stress and energy loss coefficient for different cycles keep nearly constant after the third loading-unloading cycle. After being compressed for 10 cycles, the volume of the GA remains almost the same without a discernible diminish or cracking compared to the original morphology, retaining 84% of the maximum stress with 5% of compressive strain lost. It is noted that the GA exhibits excellent energy absorption or dissipation capability, which is a key function in cellular materials^34^. The energy loss coefficient in the first cycle is 86.7% and can maintain a value higher than 83% during the next nine cycles, which is superior to all the reported GAs. For example, Qiu and coworkers reported that the GA with a density of 3 mg cm^−3^ had energy loss coefficient 75% in the first cycle and 63% in the second cycle^12^, while those for the cork-like GA with a density of 5.1 mg cm^−3^ decreased from ~82% to ~60% for the second cycle^22^.

The high degree of recoverable deformation of the as-synthesized GA can be attributed to its cellular structure and the tortuous walls. The well-organized cell structures with tightly bridged walls (Fig. 1b–d) can maximize mechanical strength^20^,^35^. Each cell can share the stress because of the completely interconnected cells in the monolith, which significantly contributes to the excellent elastic behaviour versus the severe deformation. Meanwhile, the tightly packed multilayered graphene sheets derived from the strong π-π interactions greatly enhance the strength and elastic stiffness for the wall^36^. At the high deformation state of the monolith, it is beneficial to the recovery of the closer opposing wall controlled by van der Waals adhesion. The ultrathin wall of the GA (Fig. 1e) is favorable for increasing the connection points and the stress-bearing in the out-of-plane direction^12^,^21^,^22^,^34^, which contributes to the elastic bending stiffness. The formed wrinkles and scrolls on the wall of the GA (Figs 1e, 2g–i) can further enhance the elastic stiffness^37^,^38^,^39^. For the energy dissipation capability of the GA, firstly, the microroughness and stacking configuration of graphene sheets in the cell walls maximize the contact area, which can generate a higher degree of friction between flakes during buckling and recovering. The higher energy dissipation coefficient with outstanding cyclic stability may be attributed to the appropriate reduction degree of GA. It has been found that a higher degree of reduction for cellular graphene monolith leads to the less dissipates energy, because the higher crystallized reduced GO undergoes less damage during cycling and consequently dissipates less energy^22^.

### Oil absorption capabilities of GA

The combination of the extremely low density, super high porosity and excellent elasticity makes the as-synthesized GA an attractive candidate for absorbing and desorbing organic liquids. As shown in Fig. 3c, the GA filled with n-hexane can be compressed above 90% of its initial height and the majority of the liquid can be squeezed out easily. Then the compressed structure of GA is recovered completely after absorbing the extrusive oil again. The process for oil absorption and release was repeated for dozens of times and the absorption capability maintained above 90%. The n-hexane absorption capacity (Q) of GA-3 was compared with those of different graphene based aerogels (Fig. 3d). The Q value of GA-3 can reach 226, which is significantly higher than the reported values for various graphene spongy materials, including graphene sponge fabricated by thermally reducing the monolith derived from freeze-drying GO suspension (Q = 125)^7^, graphene sponge fabricated using hydrothermal treatment with the assistance of thiourea (Q = 75)^40^, graphene sponge fabricated using hydrothermal treatment (Q = 44)^41^ and graphene foams fabricated by leavening strategy (Q = 36)^11^. More importantly, there is almost no loss of absorption capability after the GA was used for ten absorption-release cycles (inset of Fig. 3d).

### GA as a reactor for catalytic hydrogenation

**Figure 4:**
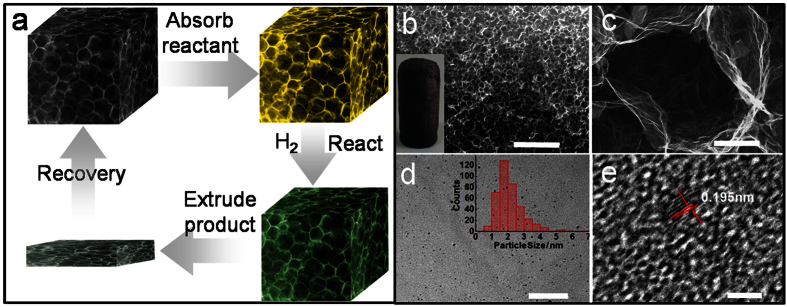
Scheme for the catalytic hydrogenation in Pd/GA and characterizations for Pd/GA-3. (**a**) The illustration of catalytic hydrogenation using GA as a reactor. (**b**) SEM image of the Pd/GA, the inset shows the photograph of the Pd/GA monolith with an integral structure. (**c**) Magnified SEM image of cell structure in Pd/GA. (**d**) TEM image of graphene sheet of the wall bearing Pd nanoparticles. The inset shows the size distribution of Pd nanoparticles. (**e**) HRTEM image of Pd/GA reveals the lattice spacing of about 0.195 nm, corresponding to the lattice fringe of Pd (2 0 0). Scale bars, 100 μm (**b**), 3 μm (**c**), 20 nm (**d**), and 2 nm (**e**), respectively.

The graphene based materials have been widely used as catalyst support for heterogeneous reactions^42^. Here the as-synthesized GA was used as an appealing fixed-bed reactor for organic reactions by taking account of its unique features. First, the super porosity endows it with high absorption capability for organic reactants; second, the interconnected macroporous structure facilitates the diffusion kinetic of catalytic reaction; third, the highly compressible feature allows the easy separation of product and the regeneration of GA. The route for the catalytic hydrogenation using GA as a reactor is illustrated in Fig. 4a. Firstly, the Pd-loaded GA was prepared *in situ* by the similar route to the GA fabrication, where potassium tetrachloropalladate was introduced to the GO dispersion. The as-synthesized Pd/GA was immersed in organic liquid to absorb the sufficient amount of reactant. Then the reaction proceeded under the drive of hydrogen. After the reaction, the products were separated from Pd/GA by simply squeezing the liquids out. The Pd/GA monolith can be directly recovered by absorbing the reactant for the next circular reactions.

**Figure 5:**
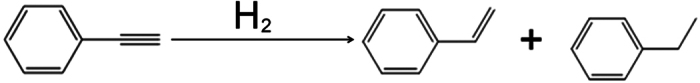
Hydrogenation of phenylacetylene.

The Pd/GA monolith presents an ordered macroporous network (Fig. 4b,c), similar to that of the pure GA shown in Fig. 1b. It indicates that the Pd loading process cannot change the cellular structure of GA, in other words, the proposed strategy shown in Fig. 1a is applicable not only for the synthesis of cellular GA but also for the fabrication of cellular GA composites (e.g. metal/GA and metal oxide/GA). The TEM image confirms that the Pd nanoparticles with an average size about 2 nm are well dispersed on GA (Fig. 4d). The nanoparticles keep the crystalline structure with lattice fringe of Pd (200)^43^, as evidenced by the high-resolution TEM (HRTEM) shown in Fig. 4e. The Pd/GA monolith has a density of 3.0 mg cm^−3^, a little higher than that of pure GA (2.8 mg cm^−3^). The Pd loading in the GA is 0.6 wt%, as determined by inductively coupled plasma atomic emission spectroscopy (ICP-AES). The catalytic activities of Pd/GA to the selective hydrogenation of phenylacetylene (Fig. 5) were studied.

At H_2_ pressure of 1 MPa, 62.3% phenylacetylene could convert within 0.5 h with >99% selectivity to styrene (entry 1, Table 2). With increasing reaction time, the conversion of phenylacetylene increases and it could almost completely convert in 1 h, with a decreased selectivity to styrene (78%, entry 3). The catalyzing process is sensitive to the hydrogen pressure, i.e. the low hydrogen pressure is not favorable for the conversion of phenylacetylene (entry 4–6). For example, the TOF value decreases significantly from 5917 h^−1^ at 1 MPa or 2 MPa to 1450 h^−1^ at 0.1 MPa. It is easily understood that since no energy input (e.g. stir) was involved in the reaction process, the diffusion of H_2_ in GA is favored at higher pressure and thus promotes the reaction rate. Qiu and coworkers have studied the catalytic activity of Pd/GA for the hydrogenation of phenylacetylene by using the conventional heterogeneous catalytic process in solvent medium^44^. At hydrogen pressure of 0.1 MPa and other similar experimental conditions, the TOF value is 60 h^−1 ^44 (entry 7), more than 23 times lower than that obtained by using GA as a reactor. For comparison, we also studied the catalytic activity of the commercial Pd/C catalyst for the hydrogenation of phenylacetylene by the similar route illustrated in Fig. 4a. The Pd/C catalyst showed no selectivity to styrene and the TOF value is much lower than that of Pd/GA (entry 8). The high catalytic activity of using GA as a reactor can be mainly attributed to its 3D interconnected macroporous structure, which facilitates the diffusion kinetic of the catalytic reaction. Also, the highly dispersed ultra-small Pd nanoparticles (~2 nm) could increase the density of catalytic active sites and accelerate the reaction^45^,^46^.

The Pd/GA monolith can be directly recovered by absorbing the reactant phenylacetylene for the next circular reaction (Supplementary Fig. 9). Only a little loss of activity was observed after the Pd/GA was used for 5 cycles (entry 9–12), which can be ascribed to the loss of Pd nanoparticles during the recovery process. The conversion of phenylacetylene is 96.7% for the fifth run, while the selectivity for styrene increases to 89.7%, indicative of the decrescent degree of further hydrogenation. The TEM image of the Pd/GA after used for five runs shows no significant particle aggregation (Supplementary Fig. 10).

## Discussion

Here we proposed a facile, easily controlled and versatile route to fabricate graphene-based cellular aerogel, by the chemical reduction of GO assemblies at oil-water interface. The as-synthesized GA has a cellular structure, in which the walls of the cell closely pack in a highly ordered manner to maximize mechanical strength. Such a cellular structure makes the GA ultralight (with density <3 mg cm^−3^) yet mechanically resilient. The properties of the GAs can be easily modulated by the concentration of GO precursors.

It is worth noting that to achieve an ordered macroporous aerogel of graphene is usually difficult, especially as GA has an ultralow weight. Here the combination of ordered macroporous structure and ultralow weight of the as-synthesized GA is due to the ingeniousness of the proposed strategy (Fig. 1a). First, the formation of GO-stabilized emulsions with very low GO concentration and large dispersed phase provide a prerequisite for the production of GA with high porosity and ultralow density. Second, the mild reduction condition of GO (70 °C) can well preserve the emulsion stability and ensure the *in situ* reduction of GO assemblies at water-oil interface, which is favorable for forming ordered macroporous structure of GA. Third, the simultaneous removal of the two liquids by freeze drying could minimize the damage to the macroporous structure of GA during the solvent separation process. The proposed route is applicable for the synthesis of cellular network of GA and GA composites (e.g. metal/GA and metal oxide/GA).

The unique features of the as-synthesized GA confer it great advantages to act as an appealing reactor for catalytic hydrogenation, e.g. the high absorption capability for organic reactants due to the super porosity, the improved mass diffusion owing to the interconnected macroporous structure and easy separation of product and the regeneration of GA resulting from the highly compressible feature. The Pd-loaded GA has exhibited exceptional catalytic activity for the selective hydrogenation of phenylacetylene. We anticipate that more GA composites can be fabricated by the proposed route and find more applications in chemical reactions in future.

## Methods

### Materials

Graphite flake (Natural, 325 mesh, 99.8%) and Pd/C (5 wt%) was supplied by Alfa Aesar. Analytical grade KMnO_4_ was purchased from Shanghai Chemical Reagents Company. Analytical grade NaNO_3_, NaHSO_3_, 99.7% cyclohexane and 98% phenylacetylene were purchased from J&K Scientific Co., Ltd. Analytical grade K_2_PdCl_4_ was produced by Acros. 98% H_2_SO_4_, 99% n-hexane, 30% H_2_O_2_ aqueous solution and deionized water were provided by Beijing Chemical Works. All these materials were used directly without further purification.

### GO synthesis

A modified Hummers method was used to prepare GO flakes by the oxidation of natural graphite^47^. Typically, a flask containing concentrated sulfuric acid (120 mL) was cooled using an ice bath. NaNO_3_ (2.5 g) and graphite flakes (5 g) were added to H_2_SO_4_ under vigorous stirring. KMnO_4_ (15 g) was slowly added to the reaction flask at room temperature. Then the flask was transferred into a water bath of 35 °C and the reaction mixture was stirred for 0.5 h until a thick paste formed. 230 mL of water was added and the reaction temperature was kept at 98 °C for 40 min. Finally, the mixture was diluted to 800 mL, followed by the slow addition of 20 mL H_2_O_2_ (30%). The yellow dispersion was filtered and washed with HCl aqueous solution (10%) to remove metal ions, followed by washing with water to remove the acid. The obtained solid was freeze-dried for 48 h to remove the residual water. GO aqueous solution was obtained by dispersing the GO flakes in water using an ultrasonic tip (UP200S, Hielscher).

### Emulsion formation and characterization

The emulsification was performed on a Shanghai Zhisun JYD-150 sonicator, which consists of a generator, a converter and a probe horn. The GO aqueous solution with NaHSO_3_ (8 mg mL^−1^) and cyclohexane were mixed in a 50 mL glass vessel, which was then submerged in water bath of 25 °C. The cyclohexane-to-water volume ratio was 1:1. The GO concentration in emulsion was 0.25, 0.75, 1.25 and 2.00 mg mL^−1^. The Ø 3 mm tip of the probe was immersed in emulsion. A 5 min sonication section was applied for the emulsification, within which a 20-second break was utilized after each 5-second sonication section. The sonic frequency was 25 KHz, with the output power at 40 W. The microstructure of the emulsion was characterized by an OLYMPUS FV1000-IX81 confocal laser scanning microscopy with the employed excitation wavelengths of 559 nm. A 5.0 uL emulsion was trickled on a 0.7 mm thick cover slip through microsyringe and covered with another, which was monitored and captured by a digital CCD.

### Fabrication of GA and characterization

The glass vessel containing the emulsion above was immersed in water bath of 70 °C for 12 h. Then the vessel was cooled to room temperature naturally and the floating gel was immersed in deionized water for 24 h to remove residual reducing agent. The graphene gel was directly frozen at −20 °C for 12 h and the two liquids were removed simultaneously by freeze drying to obtain aerogel. The morphologies of the as-synthesized aerogel were characterized by SEM (HITACHI S-4800) and TEM (JEOL-1010) operated at 100 kV. AFM measurements were performed on a tapping-mode atomic force microscope (Nanoscope IIIa, Digital Instruments, Santa Barbara, CA), with a silicon cantilever probes. XRD was performed on a Rigaku D/max-2500 diffractometer with Cu Kα radiation (λ = 1.5418 Å) at 40 kV and 200 mA. UV-visible spectrum was determined using a Varian Cary 1E UV-vis spectrophotometer. XPS was determined by VG Scientific ESCALab220i-XL spectrometer using Al Ka radiation. The porosities were determined by mercury intrusion porosimetry using a Micromeritics Autopore IV 9500 porosimeter. The sample was subjected to a pressure cycle starting at 5 psia, increasing to 44500 psia in predefined steps to give pore size/pore volume information. The compression test was carried out using a model 3342 Instron Universal Testing Machine at a rate of 50% strain min^−1^.

### Oil absorption by GA

The absorption capability of the GA was measured by immersing the sample in n-hexane. The weights before (wt_b_) and after absorption (wt_a_) were recorded and the weight uptake Q (g g^−1^) was calculated by Q = (wt_a_-wt_b_)/wt_a_. The n-hexane in GA was heated and released at 85 °C for the absorption cycles.

### Pd/GA synthesis and characterization

The Pd/GA was prepared by the route similar to the GA fabrication. The main difference is that the desired amount of K_2_PdCl_4_ was introduced to the GO aqueous dispersion, while the other experimental procedure and conditions were the same as those for GA-3 fabrication. The loading content of Pd was determined by ICP-AES (VISTA-MPX). The morphologies of Pd/GA were characterized by SEM (HITACHI S-4800), TEM (JEOL-1010) and HRTEM (JEOL-2100F).

### Catalytic test of Pd/GA

Phenylacetylene (2.0 mmol) was firstly absorbed by the Pd/GA (6 mg). The monolith was put into a 10 mL stainless steel autoclave, followed by evacuation and filling with H_2_ for three times. Subsequently, H_2_ with a suitable pressure was added to the autoclave, which was kept in a 30 °C water bath. After the desired time, the autoclave was depressurized slowly to ambient pressure. The Pd/GA was squeezed and washed with ethanol. The product was analyzed using gas chromatograph (Agilent 6820) equipped with a flame ionization detector (FID) and a PEG-20M capillary column (0.25 mm in diameter, 30 m in length).

## Additional Information

**How to cite this article**: Zhang, B. *et al*. Cellular graphene aerogel combines ultralow weight and high mechanical strength: A highly efficient reactor for catalytic hydrogenation. *Sci. Rep*. **6**, 25830; doi: 10.1038/srep25830 (2016).

## Supplementary Material

Supplementary Information

Supplementary Movie S1

## Figures and Tables

**Table 1:** Properties of the GAs synthesized at different GO concentrations.

GAs	Density/mg cm^−3^	C/O ratio	porosity/%	V_pore_/cm^3^ g^−1^	D_macro_/μm	Young’s modulus/KPa
GA-1	1.1	5.16	98.6	130.0	100	4.3
GA-2	2.2	6.16	97.2	104.1	83	8.7
GA-3	2.8	6.39	95.3	88.3	70	12.9

**Table 2:** Catalytic activities of supported Pd to the selective hydrogenation of phenylacetylene.

Entry	Catalyst	Time/h	P_H2_/MPa	Temp./^o^C	Conversion/%	Selectivity/%	TOF/h^−1^
1^a^	Pd/GA	0.5	1	30	62.3	>99	7372
2^a^	Pd/GA	0.75	1	30	82.4	94.7	6500
3^a^	Pd/GA	1	1	30	>99	78.0	5917
4^a^	Pd/GA	1	0.1	30	24.5	>99	1450
5^a^	Pd/GA	1	0.3	30	61.3	98.4	3627
6^a^	Pd/GA	1	2	30	>99	73.2	5917
7^b^	Pd/GA	1	0.1	30	75.0	97.0	60
8^c^	Pd/C	1	1	30	>99	0	710
9^d^	Pd/GA	1	1	30	97.2	89.3	5751
10^e^	Pd/GA	1	1	30	98	87.2	5798
11^f^	Pd/GA	1	1	30	97.4	88.0	5764
12^g^	Pd/GA	1	1	30	96.7	89.7	5721

^a^Pd/GA synthesized in this work (6 mg), Pd (0.338 μmol in Pd/GA), phenylacetylene (2.0 mmol), no solvent.

^b^Pd/GA catalyst reported in ref. 44, Pd (12.6 umol), ethanol (10 mL), phenylacetylene (1 mmol).

^c^Commercial Pd/C catalyst (6 mg), Pd (2.82 μmol in 5 wt% Pd/C), phenylacetylene (2.0 mmol), no solvent.

^d–g^Pd/GA was reused for the second, third, fourth and fifth runs under conditions the same with those of entry 3. Turnover number (TON) = the number of moles of the product per mole of Pd; TOF = TON h^−1^.
