# Supplementary material for: Cellular graphene aerogel combines ultralow weight and high mechanical strength: A highly efficient reactor for catalytic hydrogenation
Source: Sci Rep. 2016 May 12;6:25830. doi: 10.1038/srep25830 (PMC4865943; doi:10.1038/srep25830)
Supplement: Supplementary Information [file srep25830-s1.doc]

Supplementary materials for:

**Cellular graphene aerogel combines the ultralow weight and high mechanical strength: A highly efficient reactor for catalytic hydrogenation**

Bingxing Zhang, Jianling Zhang*, Xinxin Sang, Chengcheng Liu, Tian Luo, Li Peng, Buxing Han, Xiuniang Tan, Xue Ma, Dong Wang & Ning Zhao

Beijing National Laboratory for Molecular Sciences, CAS Key Laboratory of Colloid and Interface and Thermodynamics, Institute of Chemistry, Chinese Academy of Sciences. *e-mail: zhangjl@iccas.ac.cn


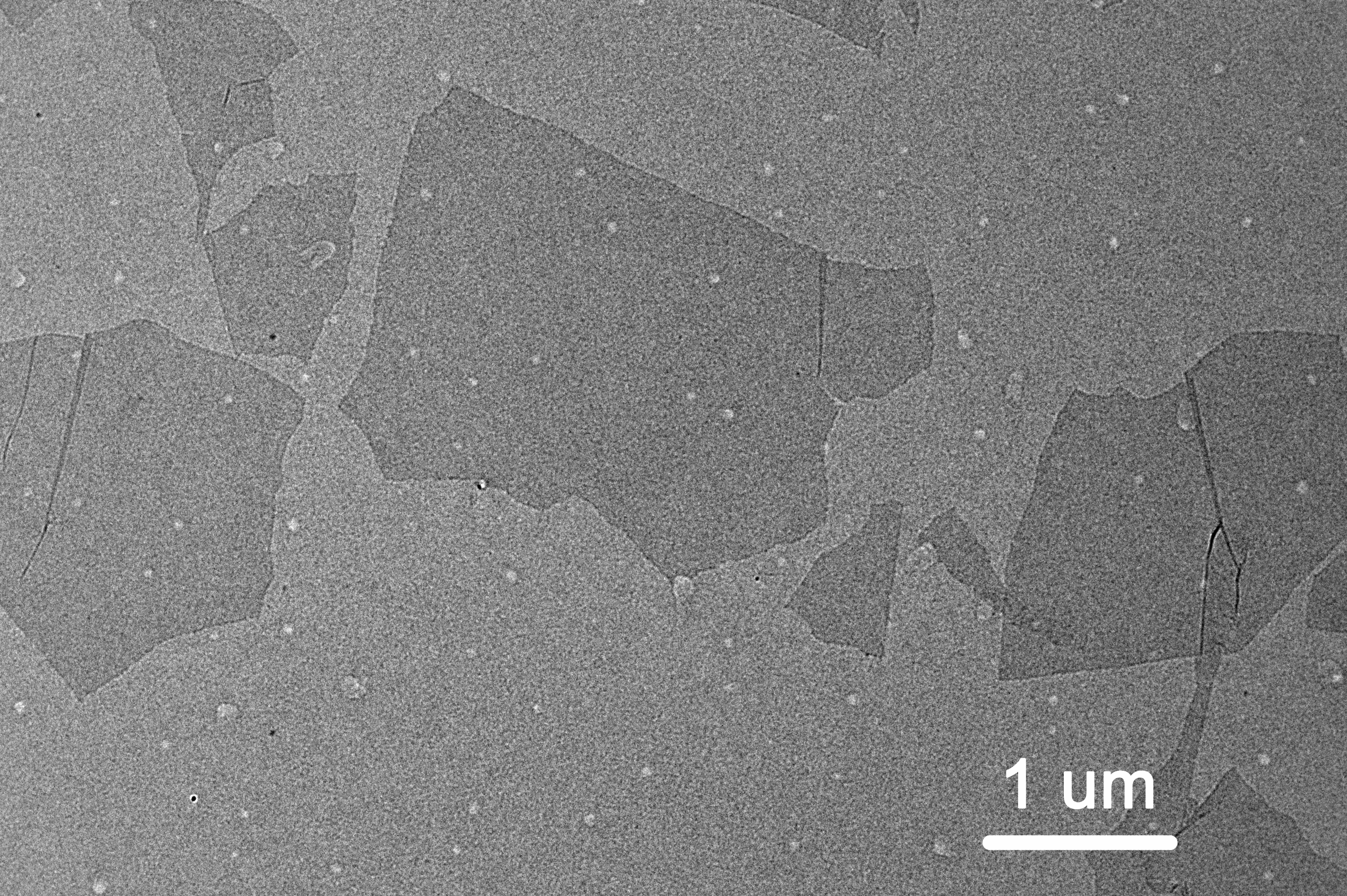

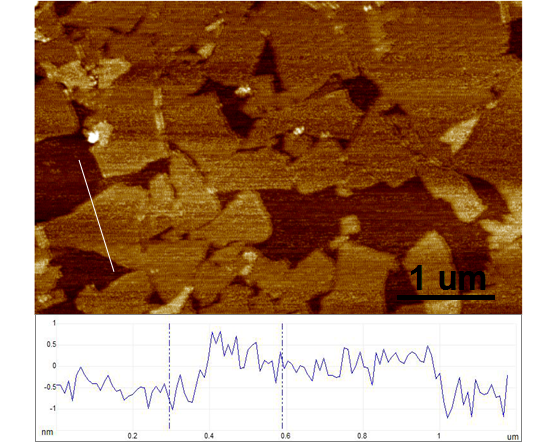


**Figure 1 |** TEM (left) and AFM (right) images of the GO flakes


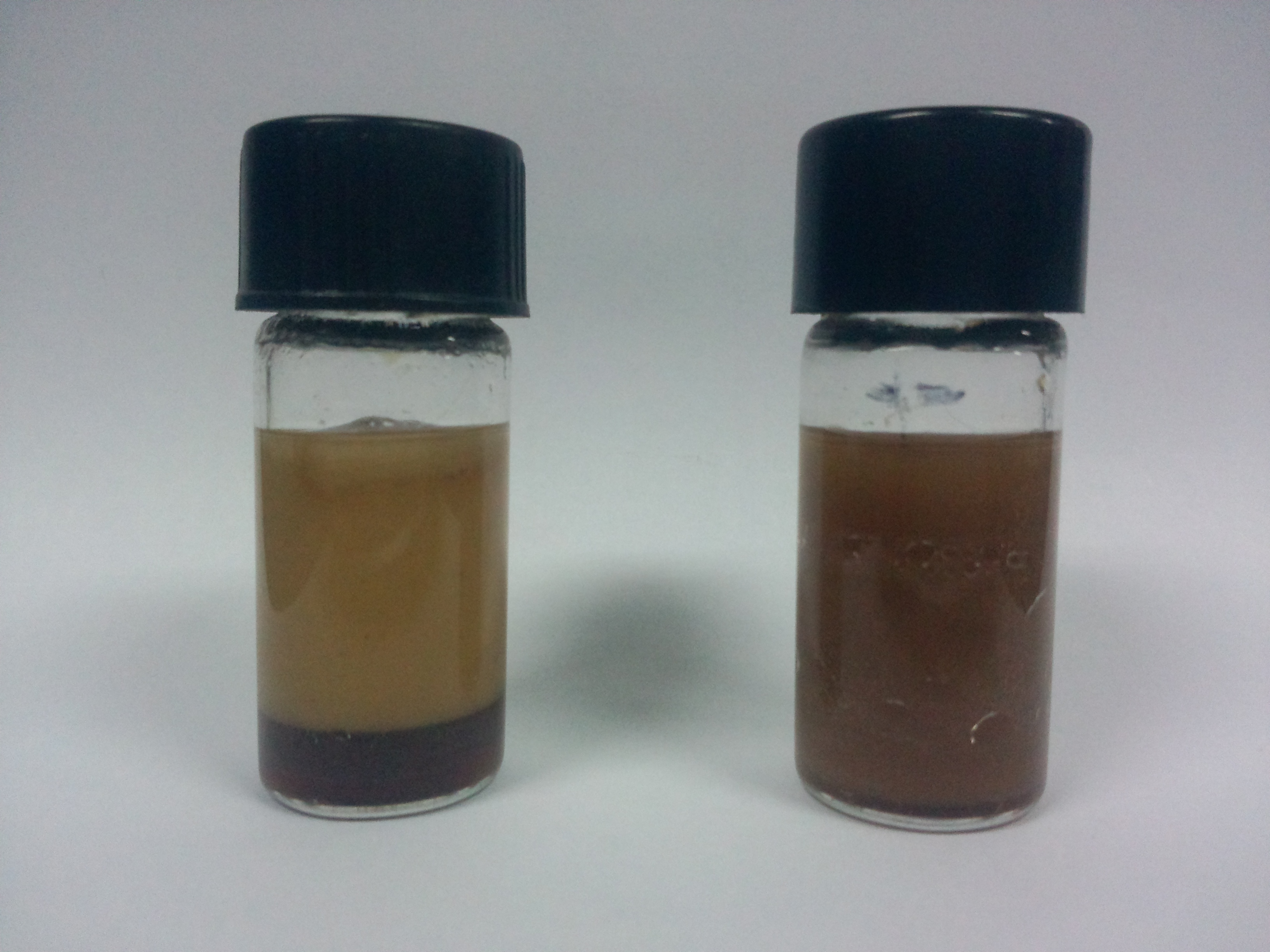


**Figure 2 |** Photographs of the GO stabilized emulsion (GO concentration: 0.75 mg mL^-1^) without (left) and with NaHSO_3_ (right), which were observed after the emulsion being formed for 30 minutes.


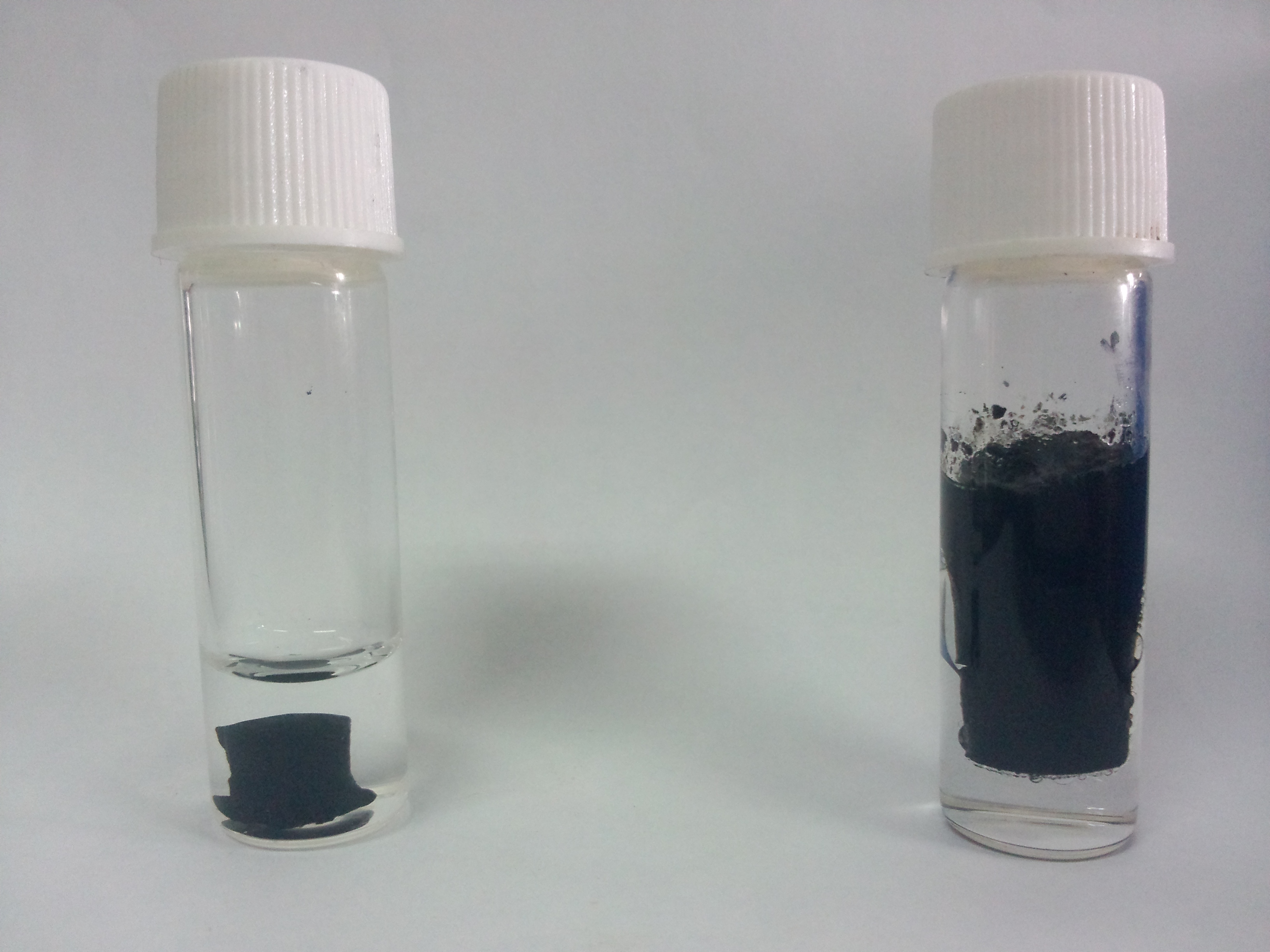

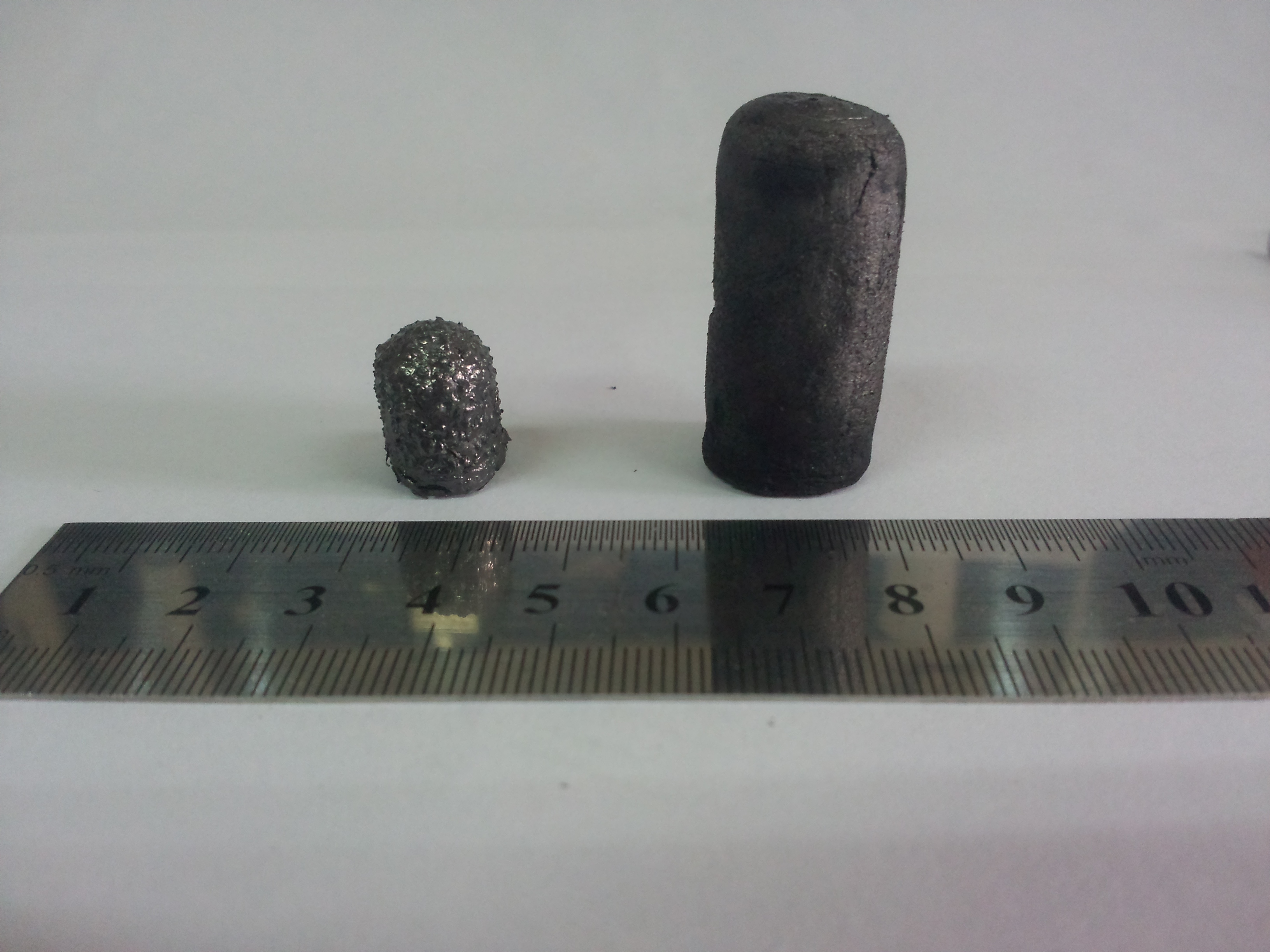



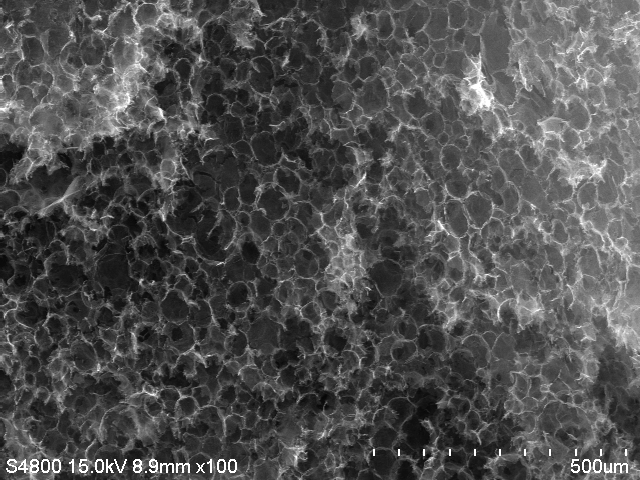


c

d

a

ba

**Figure 3 |** **a, b,** Photographs of the graphene gels (a) and GAs (b) derived from reducing GO in aqueous solution (left) and reducing GO in cyclohexane-in-water emulsion (right). **c, d,** SEM images of the GAs derived from reducing GO in aqueous solution (c) and reducing GO in cyclohexane-in-water emulsion (d).

**Figure 4 |** C 1s XPS spectra of GO. The components of carbon bonds: C=C (284.7 eV), C-C (285.6 eV), C-O (hydroxyl and epoxy, 286.8 eV), C=O (287.8 eV) and O-C=O (288.8 eV).

a


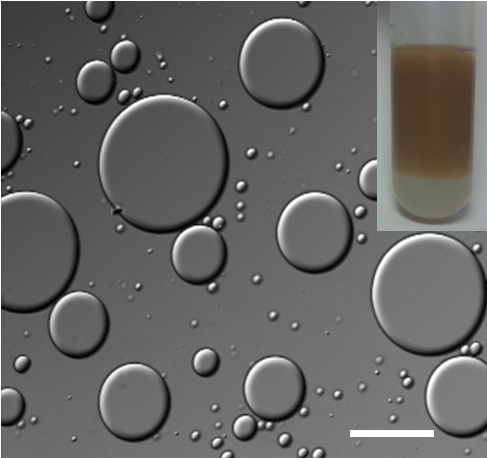

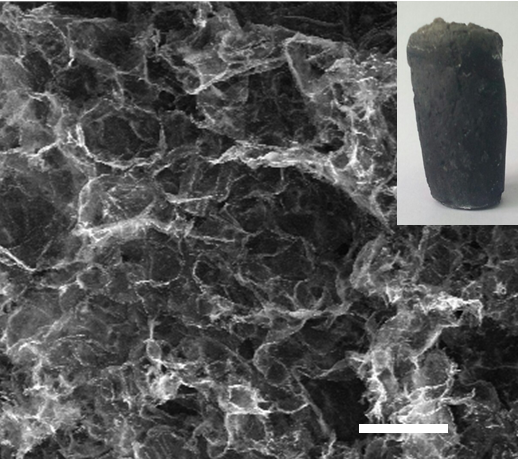


ba

**Figure 5 | a,** CLSM image of the emulsion with initial GO concentration of 0.25 mg mL^-1^. The inset shows the photograph of the emulsion. **b,** SEM image and photograph (inset) of the GA synthesized from the emulsion. Scale bar 100 μm.

**Figure 6 |** XRD patterns of GO and the GAs.

(b)

(a)

(d)

(c)

**Figure 7 |** XPS spectra of GO (a), GA-1 (b), GA-2 (c) and GA-3 (d). The atomic ratio of carbon and oxygen (C/O) can be obtained by taking the ratio of C 1s to O 1s peak areas.

**Figure 8 |** Macropore size distributions of the GAs determined by mercury porosimetry method.

**Movie 1 |** Compression test of GA-3.


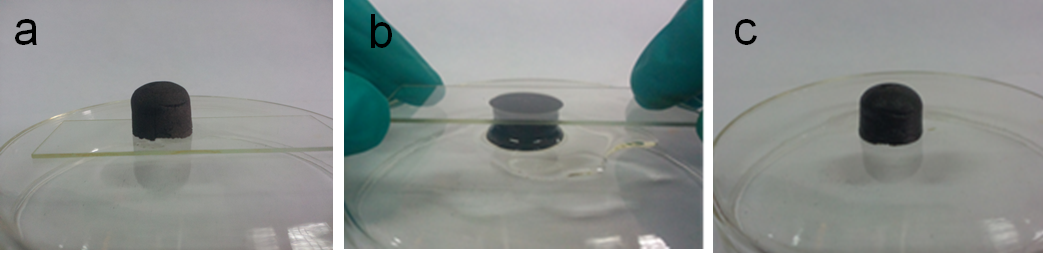


**Figure 9 |** Photographs of the Pd/GA after the first reaction (**a**), squeezing out liquids (**b**) and absorbing phenylacetylene again (**c**).


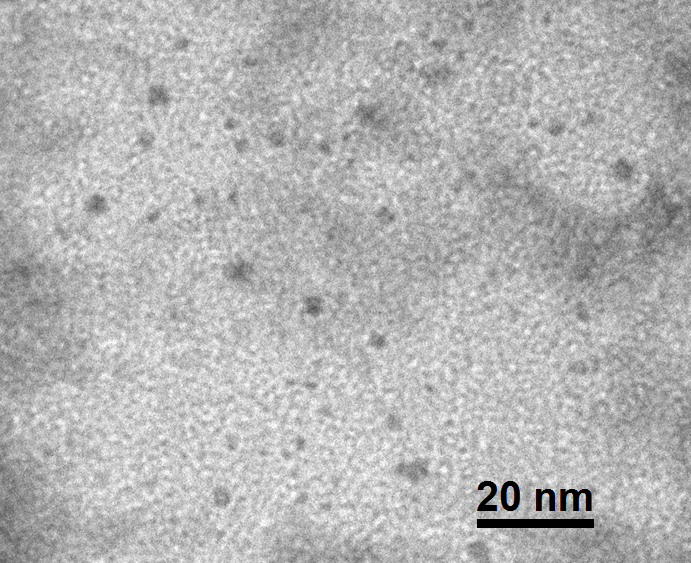


**Figure 10 |** TEM image of the Pd/GA t after used for five runs.

**Table 1.** Properties of GAs fabricated by different synthetic methods.

| Materials | Preparation  method | Additive | Density  (mg cm^-3^) | Pore sizes  (μm) | Young's  Modulus (kPa) | Ref. |
| --- | --- | --- | --- | --- | --- | --- |
| GA-3 | Chemical reduction of GO stabilized emulsion | None | 2.8 | 70 | 12.9 | This work |
| GA | directional freezing-further reduction | None | 3.25 | 100-200 | 6.73 | 21 |
| GA | Improved hydrothermal | None | 6.73 | ~100 | 16.83 | 23 |
| Spongy graphene | solvothermal | None | 1.85 | ~20 | 8.56 | 6 |
| GA | multi-step soft/hard template | PVA | 6.1 | ~65 | 5.1 | 22 |
| GA | Ethylenediamine- microwave irradiation processing | Ethylened-  iamine | 3-5 | ~100 | 16.7 | 12 |
| Carbon Aerogels | freeze-drying | Carbon nanotubes | 5.6 | ~50 | 4 | 18 |
